# Conflict among experts in health recommendations and corresponding public trust in health experts

**DOI:** 10.3389/fmed.2024.1430263

**Published:** 2024-07-26

**Authors:** Arch G. Mainous, Pooja Sharma, Lu Yin, Ting Wang, Bobbie L. Johannes, Grant Harrell

**Affiliations:** ^1^Department of Community Health and Family Medicine, University of Florida, Gainesville, FL, United States; ^2^Department of Health Services Research Management, and Policy, University of Florida, Gainesville, FL, United States; ^3^American Board of Family Medicine, Lexington, KY, United States; ^4^Department of Population Health Sciences, Geisinger, Danville, PA, United States

**Keywords:** trust, health experts, guidelines and recommendations, survey, adults

## Abstract

**Importance:**

Public trust in health experts has been decreasing leading to decreased adherence to expert recommendations.

**Objective:**

To evaluate public perceptions of conflict and uncertainty among experts in healthcare recommendations and association with decreased trust in health entities for accurate health information.

**Methods:**

Analysis of the US nationally representative Health Information National Trends Survey (HINTS 6–2022). Adults aged 18 and older were respondents to the survey (unweighted *n* = 5,842, representing 241 million adults). The main outcome was trust in doctors, scientists and government health agencies for health information. Analyses examined trust in experts with public perceptions of conflict about recommendations and changing recommendations.

**Results:**

There was high trust in doctors for health information (95%) versus 84% in scientists and 70% in government health agencies. Only 18% have high trust in the health information on social media. Respondents who felt expert recommendations change often were less likely to have high trust (65%) in government agencies compared to those who felt that the recommendations did not often change (82%) (*p* < 0.01). In logistic regressions controlling for age, sex, race, education, income and trust in social media for health information perceptions of low conflict among expert health recommendations is associated with likelihood of high trust in government health agencies (OR 2.86; 95% CI 1.96–4.15).

**Conclusion:**

The public has low trust in government health agencies and perceptions of conflict among experts over recommendations is likely playing a role in the erosion of trust in health experts.

## Introduction

Patient trust is critical to the provision of health care (1–3). Patients look to their physician to act in their best interest, provide accurate interpretation of tests and signs and symptoms, and make appropriate treatment recommendations (4). The physician is the medical expert, and patients trust the physician to provide accurate information and advice to improve their health.

Trust in government health agencies like the Food and Drug Administration (FDA) and the Centers for Disease Control and Prevention (CDC) in the US has been falling in some patient groups (5, 6). This effect of declining trust is not limited to the US. A lack of trust in government recommendations and health institutions has been observed in Europe and Asia as well (7, 8).

One factor that may be playing a role in the erosion of trust in medical experts, and in particular, government health agencies is the changing nature of healthcare treatment and screening recommendations (9). Uncertainty around recommendations and the perception that the experts are not sure about what patients should do can undermine trust in the experts (10, 11). Playing into this is that recommendations from different healthcare agencies and organizations may be in conflict and others change over time (12). This can confuse patients about what they should do. These changes may also be perceived as being the result of politics, not science, thereby undermining the confidence in the recommendations as being in the best interest of the patient (13).

On the other hand, the uncertainty associated with some of the advice from experts can be contrasted with a variety of individuals and groups spreading “pseudoscience” on social media who speak with a high degree of certainty (10). Some of the information presented on social media is in conflict with that presented by health expert agencies leading to erroneous beliefs and attitudes (14, 15). Consequently, self-assured specific opinions on social media can be contrasted with conflicts and perceived uncertainty among experts suggesting to the public that one voice is sure of the strategy and the other is not.

What is unclear is whether the public perception of conflict and uncertainty among experts in healthcare recommendations is associated with decreased trust in health entities as providers of accurate health information. This study is an examination of the relationship between perceived conflict and uncertainty in expert health recommendations and trust in different sources of health information in a nationally representative sample of US adults.

## Methods

We conducted an analysis of the National Cancer Institute’s Health Information National Trends Survey (HINTS) data, a nationwide and population-based survey [Survey Instruments | HINTS (cancer.gov)]. This study utilizes the dataset from HINTS 6 for the year 2022, collected from March through November 2022. This deidentified data is freely available to the public from the National Cancer Institute. The Individuals aged 18 or older in the civilian non-institutionalized population of the United States were included. This study was approved as exempt by the Institutional Review Board at the University of Florida.

### Variables

#### Uncertainty among experts on health recommendations

Uncertainty among experts on health recommendations was measured with two different questions. One question focused on experts changing health recommendations and the other focused on conflicting health recommendations. The study assessed changing health recommendation by the question “How often do health recommendations from experts seem to change over time?” Experts providing conflicting health recommendations was measured by the question “How often do health recommendations from experts seem to conflict or contradict one another?” The responses for both questions were categorized into two categories of high or often (“often,” “very often”) and low or not often (“never,” “rarely”).

#### Trust in medical experts

The study measured trust in medical experts from the question “In general, how much would you trust information about cancer from a doctor, government health agency, or scientists?” For each of the options, the respondents answered their amount of trust with each entity as “Not at all,” “a little,” “some” or “a lot.” For this analysis we recoded the responses to be high trust as “some” or “a lot” and low trust as “not at all” or “a little.” Attitude toward social media was measure by the question, “How much of the health information that you see on social media do you think is false or misleading?” Among those who reported using social media, the responses for that question were “None,” “a little,” “some” or “a lot.” We recoded the responses of “none” or “a little” high trust in the health information on social media while we considered the responses of “some” or “a lot” as low trust in social media information.

### Statistical analysis

We conducted data analysis using the “survey” package in R studio, which accounts for the complex survey design including stratification, clustering, and weighting. Sampling weights based on the HINTS complex sample design were used in the statistical analysis. The survey package functions of “svytable” and “svychisq” were utilized to derive nationally representative estimates of non-institutionalized US population, and chi-squared tests to assess the association between trust in medical experts and uncertainty in health recommendations. We also examined the relationship between having trust in social media for health information and trust in government health entities. The statistical significance of the tests was defined as a *p*-value of less than 0.05.

In an effort to assess the independent relationship between uncertainty and trust in the health care information from government health agencies we conducted a series of logistic regressions. The regressions included age, sex, race, total annual income, education and trust in health care information on social media along with the variables measuring uncertainty among experts (health recommendations from experts seem to change over time, health recommendations from experts conflict). Because of shared variance between the two perceived uncertainty questions we conducted three regressions. The first included both measures of perceived uncertainty, while the second only included perceived conflict among experts and the third only included the variable of perception of changes in recommendations.

## Results

The analysis was based on an unweighted sample of 5,842 representing 241,082,764 individuals. The population characteristics of adults aged 18 years or older are detailed in Table 1. The population proportion of individuals with high trust in doctors as sources of health information is 95%. The trust in scientists (84%) and government health agencies (70%) was lower than that of doctors. In terms of trust in the health information on social media, only 18% have high trust in the health information on social media based on the question of how much information you think is misleading.

**Table 1:** Population estimates for demographic characteristics of adults aged ≥ 18 years, 2022 (unweighted *N* = 5,842; weighted *N* = 241,082,764).

Factors	Population proportion (%)
Unweighted sample size	5,842
Weighted population size	241,082,764
**Sex (%)**	
Male	49.24
Female	50.76
**Age (%)**	
18–39 years	32.65
40–59 years	36.53
60–79 years	25.87
70 and above	4.95
**Race/ethnicity (%)**	
Non-Hispanic White	59.24
Non-Hispanic Black	10.64
Hispanic	16.26
Other	13.86
**Education (%)**	
Less than high school	7.46
High school and above	92.54
**Total annual income (%)**	
Less than $20,000	14.34
$20,000–$90,999	31.41
$100,000 and above	54.25

Tables 2, 3 represent the outcomes of a bivariate analysis investigating the relationship between trust in medical experts- doctors, scientists and government agencies and individuals’ perspectives on health care recommendations changing over time, and contradictory recommendations, respectively. Trust in doctors as sources of health information was high and was not significantly related to the respondent’s perspective on either changes in health care recommendations or conflicting recommendations. However, trust in both scientists and government agencies for health information was significantly related to both of the questions relevant to perceptions of uncertainty among health experts and health recommendations.

**Table 2:** Relationship between perceived uncertainty in experts health recommendations in different types of health information sources.

Trust in information source	Recommendations change-Often (%)	Recommendations change-Not often (%)	*p*
Doctors	94.87	94.22	0.64
Scientists	83.58	86.48	0.04
Government	67.56	75.95	0.01

**Table 3:** Relationship between perceived uncertainty in trust in different types of health information sources.

Trust in information source	Conflicting recommendations-Often (%)	Conflicting recommendations-Not often (%)	*p*
Doctors	99.44	94.69	0.97
Scientists	82.65	86.9	0.02
Government	61.44	82.02	0.01

On the other hand, as shown in Table 4, belief in the truthfulness of social media for health information had no significant relationship with trust in government health agencies as health information sources. Among individuals who have high trust in social media as an information source, 74% have high trust in government as an information source, while among people who have low trust in social media, 69% believe that government is a trustworthy source of health information (*p* = 0.11).

**Table 4:** Relationship between perceived uncertainty of social media in different types of health information sources.

Trust in information source	Misleading social media-Often (%)	Misleading social media-Not often (%)	*p*
Doctors	95.27	92.27	0.02
Scientists	85.46	82.96	0.07
Government	69.14	73.69	0.11

Table 5 provides the results of the multivariate analysis predicting high trust in government health agencies as a source for health care information. As with the bivariate analyses, low perceived uncertainty in health care recommendations from experts was associated with high trust in government health agencies. In particular, perceptions around conflict in experts opinions had the strongest relationship with trust in government health agencies as a source for health care information. The question about experts changing health recommendations has a significant relationship with trust in government as a source of health information only when it is the sole indicator of perceived uncertainty among experts.

**Table 5:** Logistic regression results of the association with high trust in government health agencies as a source of health information.

	Odds ratios (95% CI)		
	Model 1	Model 2*	Model 3^
Perception that changes in expert recommendations are not often	0.95 (0.62, 1.46)	–	1.55 (1.08, 2.24)
Perception of conflict among experts in recommendations is not often	2.86 (1.96, 4.15)	2.81 (1.98, 3.98)	–

Controls for age, sex, race, education, income, and trust in social media for health information.

Model 1—Includes both perception of changes in health care recommendation and perception of conflict in health care recommendations.

*Model 2—Includes only perception of conflict in health care recommendation.

^Model 3—Includes only perception of changes in health care recommendations.

## Discussion

This study investigated the relationship between the public perception of conflict and uncertainty among experts around health recommendations and trust in various sources of health information among a nationally representative sample of US adults. The findings underscore the high level of stable trust in doctors as sources of health information and the importance of consistency and clarity in health messaging, especially from authoritative sources such as government health agencies and scientists.

The high level of trust in doctors as sources of health information, compared to lower trust levels in scientists and government health agencies, suggests that personal relationships and direct interactions with healthcare providers play a crucial role in establishing trust. More importantly, this trust is not impacted by the perceived uncertainty. This is significant given the plethora of misinformation readily available through social media platforms, suggesting that trust in doctors may minimize the impact of personal narratives without a sound scientific basis (7). This effect has been found in multiple countries. Our finding is consistent with previous literature that has highlighted the critical role of the physician-patient relationship in fostering trust and confidence in health recommendations (4).

The significant relationship between perceived uncertainty among health experts on their recommendations and reduced trust in scientists and in particular, government health agencies is noteworthy. As might be expected, if the public perceives that the experts do not seem to be confident in their knowledge, trust in experts is correspondingly reduced. Conflicting advice from experts can also create confusion and skepticism about the motivations behind health recommendations.

The implications of these findings are significant for public health communication strategies. To maintain and enhance public trust, it is crucial for health scientists and government health agencies to communicate health recommendations in a clear, consistent, and transparent manner. Efforts should be made to explain the rationale behind changes in health guidance and to address any perceived conflicts in recommendations directly. Given the intensity of partisan political divides that characterize modern American discourse and the movement away from “traditional” sources of information for a variety subject matters, it is unsurprising that trust in governmental health agencies ranked the lowest in our analysis (16). The doctor-patient relationship exists on some levels as a personal relationship that only functions properly in a context of high trust. Furthermore, it will likely be increasingly important for doctors to interpret expert recommendations for patients, particularly when the science is particularly unsettled (4, 17). The COVID-19 pandemic undoubtedly accelerated the public’s level of distrust in the government and deepened political chasms on health-related topics (18).

Important differences and similarities between doctors, scientists, institutions, and social media should be noted. Doctors trust scientists to provide evidence-based conclusions that help inform their clinical practice. Government institutions also rely on scientists for their expertise in data analysis to develop guidelines and recommendations that will help to inform clinical practice. Among doctors, scientists, and institutions there are systems and policies in place to ensure information is accurate, such as peer review. The speed at which information can travel through social media makes it difficult to ensure that the information is valid through the same systems and policies relied upon by doctors, scientists, and institutions.

There are several limitations to our study. First, the question about trust in sources for accurate health information was asked in the context of information about cancer. Although there may be some specific attitudes related to cancer information that may not be reflected if the question were focused on another disease like diabetes it is important that the respondents framed their responses around a specific disease. This allowed them to think about information about a disease rather than a more general assessment of “health information” which would likely be too broad. Moreover, attitudes toward preventive services like vaccinations, which became particularly controversial during COVID-19 may be different. Second, the HINTS 6–2022 survey used two modes of data collection offering respondents a paper survey or an Internet option. This may have affected responses. Third, the cross-sectional design inhibits causal inference. Fourth, although decreased trust has been found in multiple countries including both Europe and Asia, our findings regarding government health care entities and their recommendations may not be generalizable outside of the US. Fifth, it is possible that some people may have higher or lower trust in the health care information based on the specific age/sex of the experts. Unfortunately, the HINTS survey did not include any questions that would indicate the age or sex of the experts.

## Conclusion

In conclusion, this study highlights the critical role of perceived uncertainty among experts making recommendations and conflicting information in shaping trust in health information sources. Addressing this issue through consistent, clear, and transparent communication and emphasizing physician-patient relationship is essential for maintaining public trust in health experts and ensuring the effective dissemination of health recommendations.

## Data availability statement

Publicly available datasets were analyzed in this study. This data can be found at: Health Information National Trends Survey https://hints.cancer.gov/data/survey-instruments.aspx#H6SurvMat.

## Author contributions

AM: Conceptualization, Methodology, Supervision, Writing – original draft, Writing – review & editing. PS: Formal analysis, Methodology, Writing – review & editing. LY: Data curation, Formal analysis, Methodology, Writing – review & editing. TW: Conceptualization, Writing – review & editing. BJ: Conceptualization, Writing – review & editing. GH: Writing – review & editing.
